# Tactile angle discriminability improvement: contributions of working memory training and continuous attended sensory input

**DOI:** 10.1152/jn.00529.2021

**Published:** 2022-04-20

**Authors:** Wu Wang, Jiajia Yang, Yinghua Yu, Huazhi Li, Yulong Liu, Yiyang Yu, Jiabin Yu, Xiaoyu Tang, Jingjing Yang, Satoshi Takahashi, Yoshimichi Ejima, Jinglong Wu

**Affiliations:** ^1^School of Psychological and Cognitive Sciences, Peking University, Beijing, People’s Republic of China; ^2^Graduate School of Interdisciplinary Science and Engineering in Health Systems, Okayama University, Okayama, Japan; ^3^Section on Functional Imaging Methods, National Institute of Mental Health, Bethesda, Maryland; ^4^Graduate School of Human and Environmental Studies, Kyoto University, Kyoto, Japan; ^5^College of Information Engineering, China Jiliang University, Hangzhou, People’s Republic of China; ^6^School of Psychology, Liaoning Collaborative Innovation Center of Children and Adolescents Healthy Personality Assessment and Cultivation, Liaoning Normal University, Dalian, People’s Republic of China; ^7^School of Computer Science and Technology, Changchun University of Science and Technology, Changchun, People’s Republic of China; ^8^Beijing Institute of Technology, Beijing, People’s Republic of China

**Keywords:** continuous attended sensory input, perceptual learning, tactile angle discriminability, tactile generalization, working memory training

## Abstract

Perceptual learning is commonly assumed to enhance perception through continuous attended sensory input. However, learning is generalizable to performance in untrained stimuli and tasks. Although previous studies have observed a possible generalization effect across tasks as a result of working memory (WM) training, comparisons of the contributions of WM training and continuous attended sensory input to perceptual learning generalization are still rare. Therefore, we compared which factors contributed most to perceptual generalization and investigated which skills acquired during WM training led to tactile generalization across tasks. Here, a Braille-like dot pattern matching *n*-back WM task was used as the WM training task, with four workload levels (0, 1, 2, and 3-back levels). A tactile angle discrimination (TAD) task was used as a pre- and posttest to assess improvements in tactile perception. Between tests, four subject groups were randomly assigned to four different workload *n*-back tasks to consecutively complete three sessions of training. The results showed that tactile *n*-back WM training could enhance TAD performance, with the 3-back training group having the highest TAD threshold improvement rate. Furthermore, the rate of WM capacity improvement on the 3-back level across training sessions was correlated with the rate of TAD threshold improvement. These findings suggest that continuous attended sensory input and enhanced WM capacity can lead to improvements in TAD ability, and that greater improvements in WM capacity can predict greater improvements in TAD performance.

**NEW & NOTEWORTHY** Perceptual learning is not always specific to the trained task and stimuli. We demonstrate that both continuous attended sensory input and improved WM capacity can be used to enhance tactile angle discrimination (TAD) ability. Moreover, WM capacity improvement is important in generalizing the training effect to the TAD ability. These findings contribute to understanding the mechanism of perceptual learning generalization across tasks.

## Graphical abstract

**Figure:**
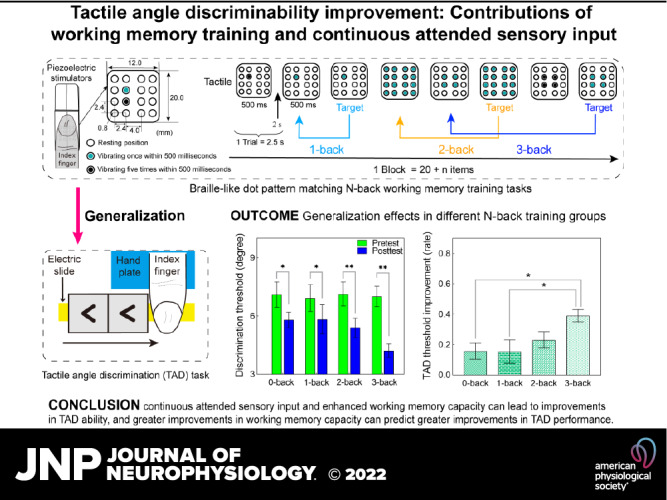

## INTRODUCTION

Perceptual learning, defined as the experience-dependent improvement of sensory systems to make sense of what we see or touch, is not limited to trained stimuli and tasks, but also appears in untrained stimuli and tasks (1–7). In a previous study (7), we found that training time intervals affect the early stages of learning but not the later stages (i.e., the last three training sessions). Interestingly, we also found that a different type of training task (tactile orientation discrimination) can improve tactile angle discrimination (TAD) performance, which implies that task-related working memory (WM) improvement may lead to perceptual generalization. Nevertheless, it is unclear how continuous attended sensory input and WM contribute to tactile object discrimination.

Continuous attended sensory input can improve perceptual discriminability to both trained and untrained stimuli (8–10). Indeed, when a training stimulus shares a particular feature with an untrained stimulus, learning is generalized to the untrained stimulus (3, 5, 8). For instance, exposure to horizontal bars on multiple fingers improves tactile spatial acuity (grating orientation) (5), and tactile orientation discrimination training enhances TAD ability (7). Nevertheless, the direction of generalization appears from simple stimuli to complex stimuli because simple stimulus features occupy higher sensory areas according to reverse hierarchy theory (11–13). Moreover, sensory training has been shown to modify the functional specializations of sensory cortical areas and even reweight their schemes in perceptual decision making (10, 14, 15). However, the training effect remains low level and stimulus driven (feature-based), implying that the benefits of perceptual generalization are relatively limited (3, 6).

Training task-independent higher-level processes such as WM can enhance perceptual performance across a wide range of tasks and stimuli (5–7, 16–19). Recent studies have shown WM training can improve maintenance ability, updating ability, distractor filtering efficiency, attention control, and even neural efficiency (6, 17, 20–23). These improvement abilities easily generalize to nontrained tasks with overlapping functional component processes (21, 24–28). Since TAD requires memory, comparison, and updating of angle information (29), we suggest that WM training improves maintenance and updating abilities, which easily generalize to TAD ability. Although tactile generalization across tasks has been observed in tactile perceptual training as a possible result of WM training in previous studies (5, 7), they did not discuss the weights of perceptual improvement that result from continuous attended sensory input and WM training. Therefore, our goals are to determine which factors contribute the most to perceptual learning and how WM training can improve tactile perception performance.

In the present study, we used a Braille-like dot pattern matching *n*-back task paradigm to manipulate the WM weight of the training task (30). We applied Braille-like dot patterns as stimuli in the *n*-back task, which could avoid the influence of similar sensory feature inputs on learning generalization. We set four different workloads with 0-, 1-, 2-, and 3-back levels. Here, the 0-back group could be regarded as a continuous attended sensory input condition to compare with the other WM training conditions. We used the TAD task applied in previous studies (29, 31, 32) as a pre- and posttest. Between tests, four subject groups were randomly assigned to four different workload (0, 1, 2, 3) *n*-back tasks to consecutively complete three sessions of *n*-back training. By comparing the TAD threshold changes between the pre- and posttest, we assessed the discrepancies among the learning effects in WM training under different workloads.

## MATERIALS AND METHODS

### Subjects

A total of 40 healthy volunteers naïve to the *n*-back paradigm of WM and the TAD task were recruited from Okayama University. Prior to the experiment, all subjects provided written informed consent in accordance with the Declaration of the Ethics Committee of Okayama University. The experiment was reviewed and approved by the Ethics Committee of Okayama University. During the experiments, the volunteers did not participate in other cognitive experiments. Each subject was randomly assigned to one of four different experimental groups: the 0-, 1-, 2-, and 3-back WM training groups. The subject attributes in each group are shown in Table 1. Each group participated in experiments over a period of five consecutive days, during which the pre- and posttests of the TAD task were performed on the first and fifth days, and different *n*-back WM trainings were conducted once each day from the second to fourth days. All the subjects completed the tasks at a given time and location.

**Table 1.** The attributes of all subjects in the four different WM training groups

	Attributes				
Groups	Number	Age Range	Means ± SD	Gender	Handedness
0-back	10	20–31 yr	23.6 ± 3.8 yr	4 female	All right-handed
1-back	10	20–25 yr	22.4 ± 1.6 yr	3 female	All right-handed
2-back	10	21–26 yr	22.5 ± 1.6 yr	4 female	9 right-handed
3-back	10	21–27 yr	22.6 ± 1.8 yr	4 female	8 right-handed

WM, working memory.

### Stimuli

#### Angle stimuli.

**Figure 1.**
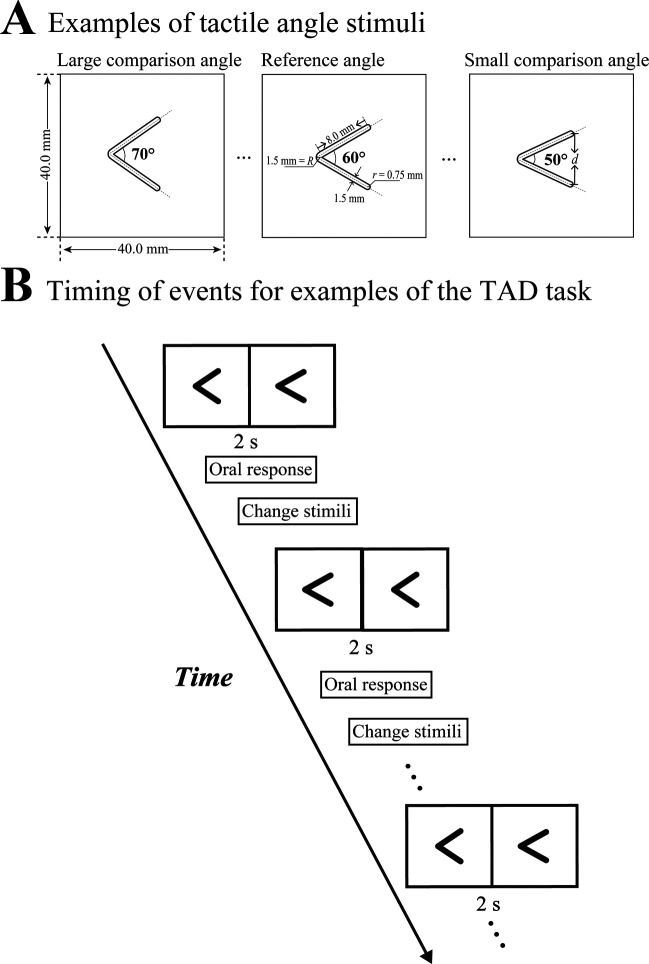
Examples of the tactile angle stimuli and tactile angle discrimination (TAD) tasks. *A*: examples of the reference angle (60°) and 2 (50° and 70°) of the 10 comparison angles applied in the TAD task. In particular, specific parameters of the angles are described. The plastic square base is 40.0 mm long and wide and 3.0 mm high. The plastic lines composing the angles are 8.0 mm long, 1.5 mm wide, and 1.0 mm high. *d* denotes the end point distance, *r* depicts the radius of curvature at the end point, and *R* represents the radius of curvature at the local apex. *B*: timing of events for examples of the TAD task. Under the control of an electronic slider, the reference angle and a comparison angle subsequently slide passively across the subject’s right index fingerpad horizontally from *left* to *right*. After perceiving the angles, the subjects are asked to orally report the larger of the two angles in one pair, or if he or she could not determine a difference, indicating that the angles were the same. The experimenter records the answer of the subject as the response data. Following that, the pairs of angles are continually changed, presented, and perceived in the same way. There are a total of 10 pairs of angles, each presented 10 times in a pseudorandom order with either the reference angle or comparison angle appearing first 50% of the time.

The tactile angles were made from a plastic polyline with two equal lines (8.0 mm long, 1.5 mm wide, and 1.0 mm high) and a plastic square base (40.0 mm long and wide, 3.0 mm high). By changing the lines symmetrically distributed along an imaginary bisector, we created 11 angles, ranging from 50° to 70°, in 2° increments, with 60° set as the reference angle and the others set as comparison angles. The angles had an accuracy of ±0.2°. Figure 1*A* illustrates examples of the reference and comparison angles in detail. The angle differences between the reference angle and the comparison angles were ±2°, ±4°, ±6°, ±8°, ±10°, with end-point distances of 7.8 and 8.2 mm, 7.5 and 8.5 mm, 7.3 and 8.7 mm, 7.0 and 8.9, and 6.8 and 9.2 mm, respectively.

#### Dot stimuli.

**Figure 2.**
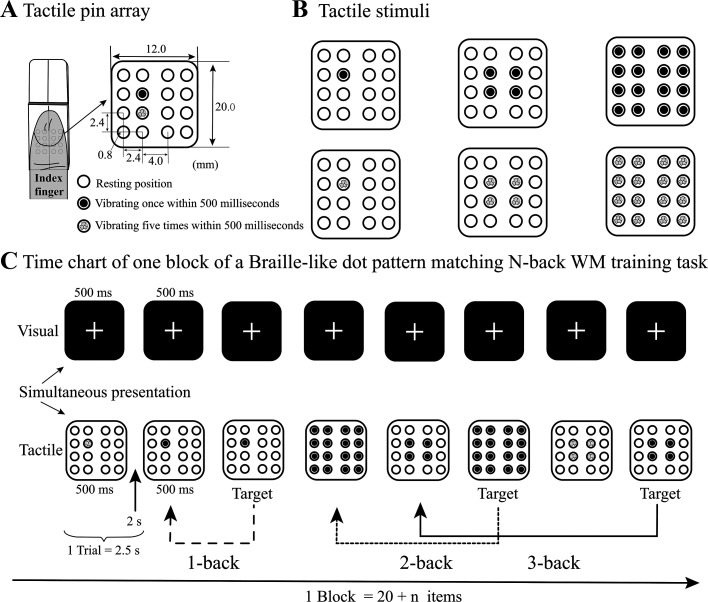
Tactile pin stimuli and examples of Braille-like dot pattern matching *n*-back working memory (WM) training tasks. *A*: tactile pin array was composed of two rows of piezoelectric Braille stimulators (KGS, Saitama, Japan). In the piezoelectric Braille stimulator, the distance between adjacent pins was 2.4 mm, and the diameter of a pin was 0.8 mm. The width and length of the pin array were 12 mm and 20 mm, respectively; the closest pin distance between the Braille stimulators was 4.0 mm; the white circle represents a pin in the resting position, the black point in the circle denotes a pin vibrating once in 500 ms, and the gray point in the circle denotes a pin vibrating five times in 500 ms. *B*: six types of tactile pin patterns were used as tactile stimuli in Braille-like dot pattern matching *n*-back training tasks. In these stimuli, one pin vibrates either once or five times within 500 ms at the center, four pins vibrate once or five times within 500 ms at the center, and all sixteen pins vibrate once or five times within 500 ms. *C*: time chart of one block of a Braille-like dot pattern matching *n*-back training task. Each tactile item was presented for 500 ms, with a white cross simultaneously presented on the center of the screen, followed by a 2000 ms interstimulus interval (ISI). The tactile items were presented to the subject’s index fingerpad, and the subject looked at the center of the screen and responded by pressing the SPACE button when the current tactile item matched the item from *n* steps earlier in the sequence. For the 0-back task, before each session, we allowed the subjects to randomly select one type of tactile dot stimulus as the target item.

Six kinds of tactile pin stimuli were used in the *n*-back tasks. We used two piezoelectric Braille stimulators (KGS, Saitama, Japan) to create a tactile pin array to present the tactile pin stimuli (Fig. 2*A*). Each stimulator consisted of eight relatively independent plastic pins, grouped in a 2 (horizontal) × 4 (vertical) array, that were elevated quickly by 0.7 mm from the rest position by a custom-built electronic drive. Thus, the tactile pin array was composed of 4 (horizontal) × 4 (vertical) pins. To be quickly recognized with the fingerpad of the right index finger, we chose three kinds of tactile pin patterns (1, 4, and 16 pins at the stimulator center) with two different vibration stimuli (vibrating once vs. 5 times within 500 ms), for a total of six types of tactile pin stimuli to be presented in *n*-back tasks. In these stimuli, 1 pin vibrates either once or five times within 500 ms at the center, 4 pins vibrate once or five times within 500 ms at the center, and all 16 pins vibrate once or five times within 500 ms (Fig. 2*B*).

### Tactile Angle Discrimination Task

We used the tactile semiautomated passive-finger angle stimulator (TSPAS) to present tactile angles to the fingerpad (34). We blindfolded the subject and sat him or her at a table with the equipment. The subjects rested their right hand on a hand plate on the table, with their right index fingerpad resting over an opening in the hand plate. Then, a pair of tactile reference angles and comparison angles subsequently slid across the stationary index fingerpad under the control of the electronic slider at a distance of 80 mm and a speed of 20 mm/s (Fig. 1*B*). The drive direction was horizontal from left to right. After perceiving the angles, the subjects were asked to orally report the larger of the two angles in one pair, or, if he or she could not determine a difference, indicating that the angles were the same. The experimenter recorded the answer of the subject as the response data. Following that, the pairs of angles are continually changed, presented, and perceived in the same way. There are a total of 10 pairs of angles, each presented 10 times in a pseudorandom order with either the reference angle or comparison angle appearing first 50% of the time.

### Braille-like Dot Pattern Matching n-Back WM Tasks

We used Braille-like dot pattern matching *n*-back WM tasks as the WM training task. One block of trials consisted of a sequence of 20 + *n* items (e.g., 20 items for 0-back, 21 items for 1-back, 22 items for 2-back, or 23 items for 3-back), with each item presented for 500 ms followed by a 2,000 ms interstimulus interval (ISI). Each block contained 14 + *n* nontargets and 6 targets that were randomly presented in the last 20 items due to the invalidation of the first *n* trials. Each session included five blocks as a training unit, including 100 + 5 × *n* items; there was a pause between blocks, and the subject could press the C button to proceed to the next block. Upon finishing one session, feedback on the accuracy of the performance was provided, and a 3-min break was required. Each training day, subjects were presented with six sessions of items (lasting ∼40–45 min as *n* changes) and were required to press the SPACE button as soon as possible whenever the current item (excluding the first *n*) was the same as that shown *n* positions back (a target); for nontargets, no responses were required. For the 0-back task, before each session, we asked the subject to randomly select one type of tactile dot stimulus as the target item. When the target item was presented, the subject was asked to press the SPACE button as soon as possible. The 0-back task rarely engaged WM; instead, it appears to be a search task. Meanwhile, it is still needed for the subjects to receive the same amount of tactile dot pattern stimulation as the other presented *n*-back tasks. Therefore, we could regard the 0-back task as a condition of continuous sensory input of tactile dot patterns, which does not contain WM. We used the Psychotoolbox-3 (33) in MATLAB (2014a, MathWorks, Natick, MA), to control the experimental procedures and stimuli presentation. During the experiment, the subject rested his or her fingerpad on the tactile pin array, and he or she looked at the center of a 17-in. color monitor. When a white cross was presented at the center of the screen, a tactile stimulus was presented simultaneously on the fingerpad, when the white cross disappeared, the tactile stimulus disappeared simultaneously (Fig. 2*C*). This cross was designed to focus the subject’s concentration.

### Data Processing and Analysis

To estimate the TAD thresholds, we used a logistic curve, as in our previous studies (7, 34, 35). The equation is as follows:

$$y=\frac{1}{1+e^{\text{β}(x-\text{α})}}.$$

It has two crucial parameters: α and β. α indicates the *x*-value of the sigmoid curve midpoint, and β denotes logistic growth. The subject’s responses were transferred to a frequency distribution, and the least square method was applied to fit a logistic curve. We defined the TAD threshold as half the difference between the angles at accuracy rates of 25% and 75%. For tactile *n*-back task performances, we used *d*′ as a measure of sensitivity, which was calculated by *Z* (hit rate) minus *Z* (false alarm rate), in which *Z* represents the inverse cumulative Gaussian distribution. Since 1 and 0 could not be transformed to *Z* values, if the hit rate was 1, we transformed it to 0.995; if the false alarm rate was 0, we transformed it to 0.005. In addition, we recorded the response time of all correct responses in all tactile *n*-back tasks.

Before conducting the analysis of variance, we used a one-sample Kolmogorov–Smirnov (K-S) and Shapiro–Wilk tests to verify that the data (see Supplemental Data; see https://doi.org/10.6084/m9.figshare.19329764) were approximately normally distributed, and a Q-Q diagram check confirmed that the data were distributed near the straight line. After that, we used the *aov* function in R programming to conduct one-way ANOVA and the *lmer* function in R programming to conduct two-way repeated-measures ANOVA and control the subject random effect resulting from the repeated measures of the pre- and posttest. Furthermore, we used the *lsmeans* function in R programming for the post hoc contrast.

## RESULTS

**Figure 3.**
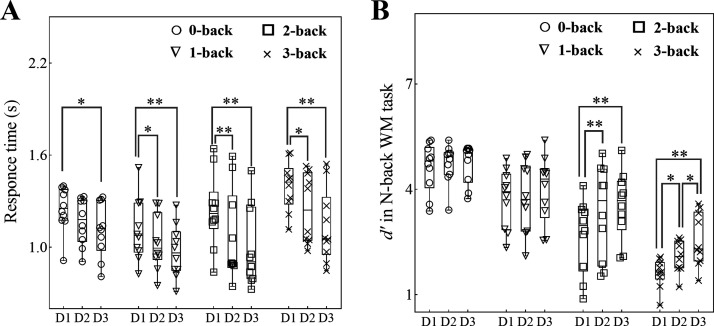
Training effects in Braille-like dot pattern matching *n*-back working memory (WM) training tasks (*n*_0-back_ = 10, *n*_1-back_ = 10, *n*_2-back_ = 10, *n*_3-back_ = 10). *A*: response time in each WM training group decreased following continuous training. In particular, the response time on the third day was significantly less than that on the first day. *B*: *d*′ = *Z* (hit rate) – *Z* (false alarm rate), *d*′s in the 0-back and 1-back WM training groups remained nearly unchanged following 3 days of training, whereas in the 2-back WM training group, *d*′ on the first day was significantly less than that on the second and third days, and *d*′ on the second day was nearly equal to that on the third day. Furthermore, in the 3-back WM training group, *d*′ significantly increased following 3 days of training; *d*′ on the third day was larger than that on the first and second days, and *d*′ on the second day was also larger than that on the first day. These results indicate that WM training with different workloads was very effective. The means and min-to-max values are shown. *P < 0.05, **P < 0.01.

First, we estimated the training effects in the Braille-like dot pattern matching *n*-back WM training groups. The response times (RTs) in each training group across three training sessions were estimated via one-way repeated-measure ANOVA. The results found that the session effects in each group were significant (0-back, *F*_2,18_ = 3.87, *P* = 0.04; 1-back, *F*_2,18_ = 12.16, *P* < 0.001; 2-back, *F*_2,18_ = 14.91, *P* < 0.001; 3-back, *F*_2,18_ = 11.80, *P* < 0.001). The post hoc comparison [Tukey’s honestly significant difference (HSD)] found that, in the 0-back training group, the RTs on the third day were significantly less than those on the first day (*t*_18_ = 2.77, *P* = 0.032); in the 1-back training group, the RTs on the second and third day were significantly less than those on the first day (*t*_18_ = 2.87, *P* = 0.026 and *t*_18_ = 4.91, *P* < 0.001, respectively); in the 2-back training group, the RTs on the second and third day were significantly less than those on the first day (*t*_18_ = 3.80, *P* < 0.01 and *t*_18_ = 5.30, *P* < 0.001, respectively); and in the 3-back training group, the RTs on the second and third day were significantly less than those on the first day (*t*_18_ = 2.67, *P* = 0.040 and *t*_18_ = 4.85, *P* < 0.001, respectively). No other significant results were found (Fig. 3*A*).

Then, the *d*′ values [*Z* (hit rate) – *Z* (false alarm rate)] in each training group across three training sessions were estimated via one-way repeated-measure ANOVA. The results found that the session effects in the 2-back and 3-back training groups were significant (*F*_2,18_ = 11.16, *P* < 0.001 and *F*_2,18_ = 19.47, *P* < 0.0001, respectively). The post hoc comparison (Tukey’s HSD) found that, in the 2-back training group, the *d*′ values on the second and third day were significantly larger than that on the first day (*t*_18_ = 3.51, *P* < 0.01 and *t*_18_ = 4.50, *P* < 0.001, respectively); in the 3-back training group, the *d*′ values on the second and third day were significantly larger than that on the first day (*t*_18_ = 3.09, *P* = 0.017 and *t*_18_ = 6.24, *P* < 0.0001, respectively) and *d*′ on the third day was significantly larger than that on the second day (*t*_18_ = 3.15, *P* = 0.014). No other significant results were found (Fig. 3*B*).

**Figure 4.**
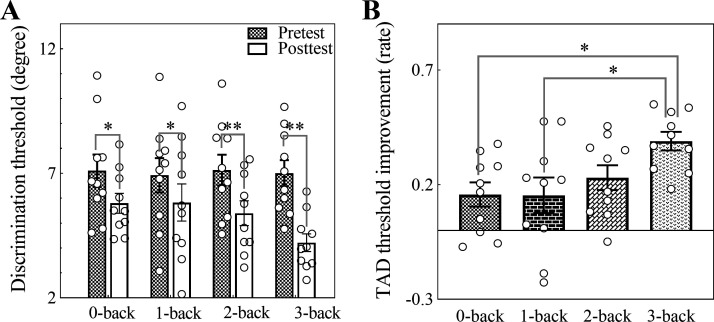
Learning effects in the different workload working memory (WM) training groups (*n*_0-back_ = 10, *n*_1-back_ = 10, *n*_2-back_ = 10, *n*_3-back_ = 10). *A*: comparison of pre- and posttest scores in the different training groups; pretest scores were significantly higher than posttest scores in the *n*-back training groups. *B*: comparison of the tactile angle discrimination (TAD) threshold improvement rate in different training groups; the TAD threshold improvement rate is defined as (pretest – posttest)/pretest in the TAD task; the TAD threshold improvement rate in the 3-back WM training group was higher than that in the 0-back and 1-back training groups. The means and standard errors (SEs) are shown. Hollow circles represent individual points in each group. *P < 0.05, **P < 0.01.

To better verify the learning effects resulting from the different workload WM trainings, we ran a 2 (testing: pretest and posttest) × 4 (training regime: 0-back vs. 1-back vs. 2-back vs. 3-back) repeated-measures ANOVA, with the TAD threshold as the dependent measure. We observed a significant main effect of testing (*F*_1,40_ = 55.93, *P* < 0.001). Importantly, we also found a marginally significant testing × training regime interaction effect (*F*_3,40_ = 2.65, *P* = 0.062). A simple interaction analysis (Tukey’s HSD) indicated that the posttest scores were lower than the pretest scores in the *n*-back WM training groups (*t*_44.4_ = 2.68, *P* < 0.05; *t*_44.4_ = 2.25, *P* < 0.05; *t*_44.4_ = 3.55, *P* < 0.01; *t*_44.4_ = 5.72, *P* < 0.0001), the pretest scores among the different *n*-back WM training groups were nearly equal, and the posttest scores among the different *n*-back WM training groups were not significantly different [*t*_63.2 (3-0 backs)_ = 1.95, *P* = 0.22; *t*_63.2 (3-1 backs)_ = 1.99, *P* = 0.20; *t*_63.2 (3-2 backs)_ = 1.46, *P* = 0.47; *t*_63.2 (2-0 backs)_ = 0.49, *P* = 0.96; *t*_63.2 (2-1 backs)_ = 0.53, *P* = 0.95; *t*_63.2 (1-0 backs)_ = 0.035, *P* = 1.00] (Fig. 4*A*). Then, to further clarify the discrepancy of the generalization effects in the different workload WM training groups, the TAD threshold improvement rate [(pretest – posttest)/pretest] was estimated via one-way ANOVA, with the results indicating that different workload WM training effects were significant (*F*_3,36_ = 3.66, *P* < 0.05). A post hoc comparison (Tukey’s HSD) further indicated that the TAD threshold improvement rate in the 3-back training group was higher than that in the 0-back and 1-back training groups (*t*_36_ = 2.85, *P* < 0.05; *t*_36_ = 2.89, *P* < 0.05) (Fig. 4*B*), and the other comparisons were not significant.

**Figure 5.**
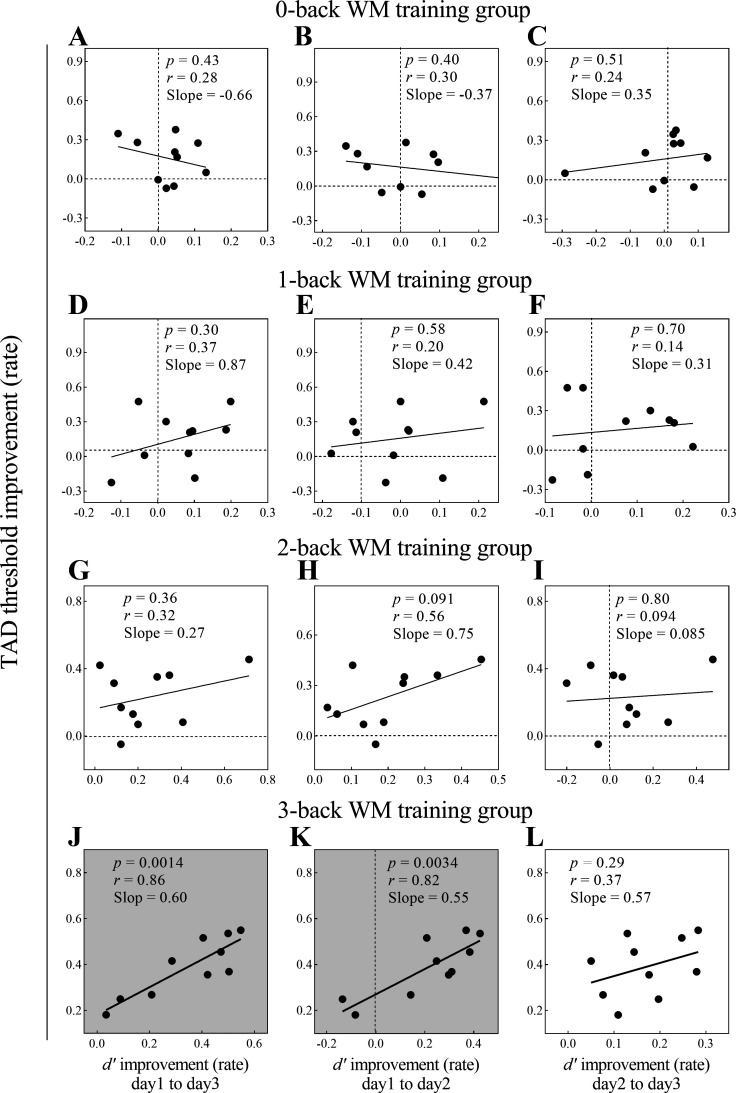
Linear prediction models in the *n*-back working memory (WM) training groups (*n*_0-back_ = 10, *n*_1-back_ = 10, *n*_2-back_ = 10, *n*_3-back_ = 10). *A*–*I*: in the 0-, 1-, 2-back WM training groups, there was no significant prediction of *d*′ improvement {*day 1* to *day 3* [(3rd *d*′ – 1st *d*′)/1st *d*′], *day 1* to *day 2* [(2nd *d*′ – 1st *d*′)/1st *d*′], or *day 2* to *day 3* [(3rd *d*′ – 2nd *d*′)/2nd *d*′]) to tactile angle discrimination (TAD) threshold improvement rate [(pretest – posttest)/pretest]. *J*–*L*: in the 3-back WM training group, the *d*′ improvement from *day 1* to *day 3* predicted the TAD threshold improvement rate with a significant fit (*P* < 0.05), and the model intercept differed significantly from 0 [*P* = 0.006, 95% confidence interval (CI): 0.068–0.29]; The *d*′ improvement from *day 1* to *day 2* [(2nd *d*′ – 1st *d*′)/1st *d*′] also predicted the TAD threshold improvement rate with a significant fit (*P* < 0.05), and the model intercept differed significantly from 0 (*P* < 0.001, 95% CI: 0.18–0.36); the *d*′ improvement from *day 2* to *day 3* [(3rd *d*′ – 2nd *d*′)/2nd *d*′] could not predict the TAD threshold improvement rate and had a nonsignificant fit (*P* = 0.29). The light gray background indicates that the linear prediction model is significant (*P* < 0.05).

To further determine whether subjects with better learning rates [(3rd *d*′ – 1st *d*′)/1st *d*′; (2nd *d*′ – 1st *d*′)/1st *d*′; (3rd *d*′ – 2nd *d*′)/2nd *d*′] in the *n*-back WM training task also showed a higher learning rate [(pretest – posttest)/pretest] in the TAD task, we ran 12 linear regressions using SPSS (SPSS Statistics, v. 22.0; IBM), with the TAD threshold improvement as a function of *d*′ improvement. The results showed that, in the 0-, 1-, 2-back WM training groups, there was no significant prediction of *d*′ improvement (*day 1* to *day 3*, *day 1* to *day 2*, or *day 2* to *day 3*) to TAD threshold improvement rate (Fig. 5, *A*–*I*); only in the 3-back WM training group, *d*′ improvements (*day 1* to *day 3* and *day 1* to *day 2*) did predict TAD threshold improvements with a significant fit (*P* = 0. 0014 and *P* = 0. 0034, respectively) (Fig. 5, *J* and *K*), and the intercepts of the models differed significantly from zero [*P* = 0.006, 95% confidence interval (CI): 0.068–0.29; *P* < 0.001, 95% CI: 0.18–0.36, respectively], but the linear model prediction of *d*′ improvement (*day 2* to *day 3*) to TAD threshold improvement was not significant (*P* = 0. 29) (Fig. 5*L*). These findings indicate that TAD threshold improvement could benefit from not only WM training but also continuous attended sensory input in the 3-back WM training group. Specifically, after one day of consolidation, the WM capacity could be improved on the second day, and WM capacity improvement could predict the TAD threshold improvement; the WM capacity acquired better improvement on the third day, and WM capacity improvement could still predict the TAD threshold improvement. These predictions suggest that the acquired WM capacity improvement had been carefully processed and consolidated, which could generalize to other tasks with similar overlapping functional components such as updating and maintenance (6, 30).

## DISCUSSION

We investigated whether tactile continuous attended sensory input and WM training could improve tactile angle discriminability, as well as how TAD ability could benefit from different workload WM trainings. Our findings showed that tactile continuous attended sensory input and Braille-like dot pattern matching *n*-back WM training could enhance TAD ability (Fig. 4*A*), that TAD threshold improvement was the best in 3-back WM training (Fig. 4*B*), and that the training effects on the 3-back WM training group could proportionally scale across TAD task performance improvement (Fig. 5, *J* and *K*). These findings suggest that while both continuous attended sensory input and improved WM capacity could generalize to TAD ability, improved WM capacity plays a larger role than continuous attended sensory input in the generalization of the training effect to the TAD ability. This suggestion could contribute to a better understanding of the mechanism of perceptual learning generalization across tactile tasks.

We found that TAD ability could be enhanced following Braille-like dot pattern matching *n*-back WM training (Fig. 4*A*). One straightforward explanation for the results is that continuous attended Braille-like dot patterns may enhance spatial acuity in the fingerpad. Although it is unclear whether the enhancement in tactile spatial acuity is generalizable to all tactile stimuli, we suggest that tactile pin stimulus training might facilitate tactile angle processing. Since tactile dot processing might be more fundamental than tactile orientation within S1, tactile pin stimulus training might benefit tactile orientation representation. Because the tactile angle consists of two different orientation polylines, the benefit may extend to tactile angle identification, which corresponds to the simple-complicated generalization principle of reverse hierarch theory (11–13). Furthermore, continuous attended tactile pinpoint contact may promote some S1-specific neural functions or increase S1 neural sensitivity (9, 36–38), which might aid in the formation of tactile angle representations processed in WM and lead to learning generalizations. Alternatively, tactile angle sensory inputs using slowly adapting afferents might converge on a shared submodality in S1 with Braille-like dot inputs that would be far more discernible in a population of slowly adapting afferents than in a population of rapidly adapting afferents (39, 40). Thus, the neural changes in the common submodality due to continuous attended Braille-like dot patterns may facilitate perceptual learning generalizations (41). In some sense, the significant intercepts in the linear prediction models might also indicate that continuous attended sensory input contributes to the generalization of the training effect to the TAD ability (Fig. 5, *J* and *K*).

Furthermore, we found that 3-back WM training resulted in the greatest improvement in TAD ability among all training groups (Fig. 4*B*). One possible explanation for this result is that improved WM capacity can generalize to TAD ability. Moreover, the linear prediction models for the 3-back WM training group further indicate that improved WM capacity plays a role in the generalization of the training effect to the TAD ability (Fig. 5, *J* and *K*). Specifically, because the *n*-back task requires continuous maintenance and updating of dynamic rehearsal items (30, 42), the higher the workload, the stronger the maintenance and updating abilities produced by the training task. Thus, 3-back WM training could significantly improve WM maintenance and updating abilities (6, 7, 20), which can be shared to higher-level cognition abilities with TAD tasks. Therefore, we suggest that improved maintenance and updating abilities generalize to TAD ability. Alternatively, since the *n*-back task requires attending the continuous tactile dot stimuli, the higher the workload, the more the attention intensity or cognitive resources required by the training task (43, 44). Thus, 3-back WM training may also induce the largest scale perceptual and neuroplastic changes in S1, which might enhance the signal-to-noise ratio and processing speed of tactile information and further result in perceptual learning generalizations (9, 38). In addition, 3-back WM training may improve central executive efficiency, prevent distraction, and enhance anti-interference ability (6, 45–48), which might allow for more mature resource deployment and contribute to TAD ability improvement.

In addition, we found that TAD ability improvements in the 0-back and 1-back training groups were nearly equal and that TAD ability improvement in the 2-back training group was between TAD ability improvements in the 1-back and 3-back training groups (Fig. 4*B*). Possible reasons for this include the fact that TAD ability improvement in the 1-back training group was most likely due to continuous attended sensory input, whereas TAD ability improvement in the 2-back training group was likely due to both continuous attended sensory input and improved WM capacity. Specifically, since the 1-back WM task in the present study is probably too easy to train WM (5, 48), the WM capacity in the 1-back training group could not be enhanced and remained nearly unchanged, i.e., the ceiling effect, although the RTs in the training task became faster following training (Fig. 3). Therefore, we suggest that continuous attended sensory input may lead to the generalization of the training effect to the TAD ability in the 1-back training group, similar to the generalization effect in the 0-back training group. Furthermore, the WM capacity in the 2-back training group improved on the second day and nearly plateaued on the third day, whereas the WM capacity in the 3-back training group was continuously enhanced following training (Fig. 3*B*); therefore, in addition to continuous attended sensory input, improved WM capacity in the 2-back training group may also result in the generalization of the training effect to the TAD ability. The generalization effect, however, was lower in the 2-back training group than in the 3-back training group. One possible reason for this is that WM training might be somewhat restricted due to the task set.

Although both high WM load training and continuous attended sensory input could improve TAD performance, high WM load training played a greater role than continuous attended sensory input (Fig. 4*B*). Sensory training might improve the processing/representation of tactile dots (e.g., improved information transmission and gain) in S1 (3, 7, 49). Furthermore, tactile dot representation possibly facilitates the processing and representation of tactile angles in the TAD task, potentially leading to learning generalizations from tactile dots to tactile angles (3, 8). However, perceptual improvement due to tactile dot exposure may result in a limited, low-level, and feature-based generalization effect. In contrast, high WM load training may improve not only the maintenance and updating ability required in the TAD task but also tactile dot representation in S1, which might facilitate tactile angle representation, integrating the two learning effects. In addition, high WM load training may modulate more attention resources to concentrate on the processes of the TAD task, avoiding interruptions and distractions as much as possible (6, 46–48). Thus, high WM load training favors high-level and top-down learning processing, which could produce a greater generalization effect than continuous attended sensory input.

This study has some broad implications for our understanding of perceptual learning. Perceptual learning generalization can result from continuous attended sensory input as well as cognitive training (such as WM training), with cognitive ability improvement playing a key role and remaining broadly applicable beyond specific stimuli and tasks. In addition, the difficulty settings in cognitive training deserve serious consideration. However, there are some limitations to the current study. At present, we could not determine which aspects of WM improvement generalize to perceptual learning, such as maintenance, updating, and central executive function; moreover, we could not determine whether there was an interaction between attended sensory input and cognitive training. Therefore, in future studies, we will focus on these questions to conduct a thorough investigation.

## SUPPLEMENTAL DATA

10.6084/m9.figshare.19329764 Supplemental data: https://doi.org/10.6084/m9.figshare.19329764.

## GRANTS

This work was supported by JSPS (Japan Society for the Promotion of Science) KAKENHI (JP18K15339, JP21H05827, and JP20K07722) and JST (Japan Science and Technology Agency) FOREST (Fusion Oriented Research for disruptive Science and Technology) Program (JPMJFR2041).

## DISCLOSURES

No conflicts of interest, financial or otherwise, are declared by the authors.

## AUTHOR CONTRIBUTIONS

W.W., J.Y., Y.Y., H.L., Y.L., Y.Y., J.Y., X.T., J.Y., S.T., Y.E., and J.W. conceived and designed research; W.W. performed experiments; W.W. analyzed data; W.W., H.L., and J.Y. interpreted results of experiments; W.W. and Y.L. prepared figures; W.W. drafted manuscript; W.W., J.Y., and X.T. edited and revised manuscript; W.W., J.Y., Y.Y., H.L., Y.L., Y.Y., J.Y., X.T., J.Y., S.T., Y.E., and J.W. approved final version of manuscript.
